# Metabolomic Profiles of Sleep-Disordered Breathing are Associated with Hypertension and Diabetes Mellitus Development: the HCHS/SOL

**DOI:** 10.21203/rs.3.rs-3171622/v1

**Published:** 2023-07-21

**Authors:** Tamar Sofer, Ying Zhang, Bing Yu, Qibin Qi, Ali Azarbarzin, Han Chen, Neomi Shah, Alberto Ramos, Phyllis Zee, Jianwen Cai, Martha Daviglus, Eric Boerwinkle, Robert Kaplan, Peter Liu, Susan Redline

**Affiliations:** Harvard University; Brigham and Women’s Hospital; Albert Einstein College of Medicine; Brigham and Women’s Hospital; The University of Texas Health Science Center at Houston; Icahn School of Medicine at Mount Sinai; University of Miami Miller School of Medicine; Northwestern University Feinberg School of Medicine; University of North Carolina; University of Illinois at Chicago; University of Texas Health Science Center at Houston; Albert Einstein College of Medicine; Lundquist Institute at Harbor-UCLA Medical Center; Brigham and Women’s Hospital

## Abstract

Sleep-disordered breathing (SDB) is a prevalent disorder characterized by recurrent episodic upper airway obstruction. In a dataset from the Hispanic Community Health Study/Study of Latinos (HCHS/SOL), we applied principal component analysis (PCA) on seven measures characterizing SDB-associated respiratory events. We estimated the association of the top two SDB PCs with serum levels of 617 metabolites, in both single-metabolite analysis, and a joint, penalized regression analysis using the least absolute shrinkage and selection operator (LASSO). Discovery analysis included n = 3,299 HCHS/SOL individuals; associations were validated in a separate dataset of n = 1,522 HCHS/SOL individuals. Seven metabolite associations with SDB PCs were discovered and replicated. Metabolite risk scores (MRSs) developed based on LASSO association results and representing metabolite signatures associated with the two SDB PCs were associated with 6-year incident hypertension and incident diabetes. MRSs have the potential to serve as biomarkers for SDB, guiding risk stratification and treatment decisions.

## Introduction

Sleep-disordered breathing (SDB) is a common yet underdiagnosed disorder. It is estimated to affect 17% and 34% of middle aged female and male individuals, respectively^1^, but diagnosed in less than 15% of individuals with clinically significant disease^2,3^. SDB is characterized by recurring episodes of complete (apneas) or partial (hypopneas) upper airway obstruction, often accompanied by oxyhemoglobin desaturation and/or sleep fragmentation. Symptoms including snoring and excessive daytime sleepiness^4^. A growing body of epidemiological studies has found that SDB associates with increased risks for vascular and metabolic diseases, including stroke, coronary heart disease, hypertension, and diabetes mellitus^5–9^.

Underlying mechanisms proposed to associate SDB with the cardiometabolic conditions include: chronic hypoxemia, particularly nightly exposures to intermittent hypoxemia and re-oxygenation^10^; dysregulated proinflammatory responses^11^; increased oxidative stress^12^, imbalanced gut microbiome^13^, hormonal imbalance^14^, among others. Emerging evidence has shown that intermittent hypoxemia, especially high frequency desaturations, modulates the inflammatory response differently from chronic sustained hypoxemia^15^. While recent work has examined specific aspects of SDB that best predict incident outcomes^16–18^, only a few studies have tried to model more complex exposures by combining multiple SDB measures together^19,20^. Various SDB measures, such as the frequency of obstructive events (e.g., Respiratory Event Index (REI)), sleep-apnea specific hypoxic burden^21^, minimum oxyhemoglobin saturation during sleep, apnea and hypopnea event duration^22^, and others, while capturing different characteristics of SDB-related physiological stressors, tend to be correlated. Given the increasing recognition of the heterogeneity and complexity of SDB^23^, indices that combine multiple measures of SDB by accounting for the correlation among them may provide powerful approaches both for studying SDB biology and for risk stratification for incident cardiometabolic outcomes.

Metabolites, reflective of the products and intermediates of metabolism, can provide biomarkers useful for disease prediction and subtyping^24^. Studying SDB-associated metabolites may yield insights into the metabolic environment of the disorder, elucidate gender differences, and suggest SDB subtypes and related molecular mechanisms involved in the progression of cardiometabolic conditions. Untargeted metabolomic profiling is the comprehensive identification and quantification of small metabolite molecules within the biological system, and has begun to be used in sleep research to understand the cellular process such as sleep/wake regulation^25,26^, as a window on peripheral molecular clocks and oscillators^27^, and to detect biomarkers of sleep restriction^28^ and neurological degeneration among patients with obstructive sleep apnea (OSA)^29^. In a recent study^30^ we identified metabolites associated with moderate to severe OSA (defined as a Respiratory Event Index [REI] > = 15) and constructed an index composed of 14 metabolites, associated with OSA cross-sectionally, in two independent datasets. Another recent study^31^ identified metabolites associated with SDB and metabolites that changed levels following SDB treatment using continuous positive airway pressure, though without multiple testing correction. Further demonstrating the potential clinical utility of untargeted metabolite profiling, prediction models incorporating metabolites outperformed clinical predictors for some conditions^32^. Thus, untargeted metabolomics may provide a unique opportunity both for the development of biomarkers for SDB, and for utilizing such biomarkers for SDB-related risk stratification: identifying patients with increased risks for other chronic diseases.

**Figure 1:**
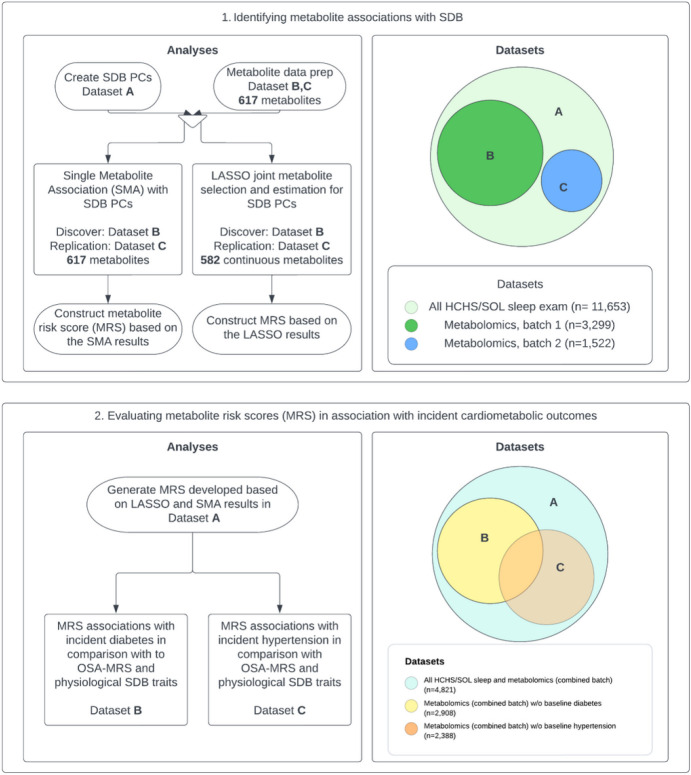
Study design diagram SDB: sleep disordered breathing; PC: principal component; LASSO: least absolute shrinkage and selection operator; OSA-MRS: metabolite risk score calculated based on coefficients from LASSO regression trained to predict OSA in previous publication (Zhang *et al.* 2022); HCHS/SOL: the Hispanic Community Health Study/Study of Latinos; baseline diabetes are based on American Diabetes Association definition (Diabetes Care 2010;33:S62–69), defined as fasting glucose >=126 mg/dL, or post-OGTT glucose >=200 mg/dL or A1C>=6.5%, or use of anti-diabetic medication; baseline hypertension is defined as systolic or diastolic BP greater than or equal to 140/90 respectively, or current use of antihypertensive medications.

We hypothesize that by combining SDB measures, and next, identifying and combining changes in their associated metabolomic environment, we can construct new SDB biomarkers that may offer additional utility compared to standard measures for identifying individuals at high risk for progression of cardiometabolic disease (Fig. 1). We use a data-driven, unsupervised principal component (PC) analysis to first construct two SDB summary measures based on several physiological phenotypes. We then study the association of the SDB PCs and the metabolic environment in a large population-based study with a high-dimensional set of measured metabolites using two methods: (1) association analysis of individual metabolites with each SDB PC, and (2) least absolute shrinkage and selection operator (LASSO) regression to identify a subset of metabolites that together best associate with SDB PCs. Based on LASSO metabolites selection and estimates, we develop SDB PC-specific metabolomic risk scores (SDB-MRS). To validate our results, we use a discovery-replication approach where we separate datasets of individuals sampled from the same target population. We then study the SDB PC-specific MRS associations with incident hypertension and diabetes mellitus.

## Methods

### The Hispanic Community Health Study/Study of Latinos

The Hispanic Community Health Study / Study of Latinos (HCHS/SOL) is a prospective community-based cohort study of 16,415 Hispanic/Latino individuals aged 18–74 years at the baseline examination (2008–2011)^33^. Individuals were selected into the study using a multi-stage stratified random sampling from four geographic regions: Bronx NY, Chicago IL, Miami FL, and San Diego CA. The sampling strategy and study design were previously described^34^. Of study participants, 12,803 individuals were genotyped. Fasting blood samples were collected at the baseline examination. Within a week of the baseline examination in the clinic, 14,440 individuals were assessed for SDB using a validated Type 3 home sleep apnea test (ARES Unicorder 5.2; B-Alert, Carlsbad, CA) that measured nasal air-flow, position, snoring, heart rate and oxyhemoglobin saturation^3^. Among the baseline HCHS/SOL participants, 11,623 returned to a second clinic visit (visit 2) from 2014 to 2017, on average 6 years after the first visit.

### Metabolomics profiling and quality control

Of HCHS/SOL participants from the baseline examination who also had genetic data, 4,004 individuals were selected at random for metabolomics profiling of fasting serum samples collected at baseline (metabolomics batch 1, processed in 2017). In 2021, additional 2,368 serum samples from 2,330 participants, also collected at baseline (see details in **Supplementary Note 1**), were profiled in a second metabolomics batch 2. Serum samples were stored at −70°C at the HCHS/SOL Core Laboratory at the University of Minnesota until analysis by Metabolon, Inc. (Durham, NC) in 2017 (batch 1) and 2021 (batch 2). Serum samples were then extracted and prepared using Metabolon’s standard solvent extraction method. Prior to extraction, samples were split into equal parts for untargeted analysis on both the gas chromatography-mass spectrometry and liquid chromatography-mass spectrometry (GC-MS and LC-MS)-based metabolomic quantification platforms^35,36^. Instrument variability was determined by calculating the median relative standard deviation (SD) for the internal standards added to each sample prior to injection into the mass spectrometers. Overall process variability was determined by calculating the median relative SD for all endogenous metabolites (i.e., non-instrument standards) present in 100% of the technical replicate samples.

We took a discovery-and-replication approach using batch 1 as the discovery and batch 2 as the replication dataset. Preprocessing of the metabolomic data is described in **Supplemental Figure S1.** First, we removed batch 2 individuals who overlapped with batch 1 and duplicated samples from the same individuals, resulting in 2,178 remaining observations. Next, we kept metabolites that were known and available in both batches and excluded xenobiotics. We also excluded metabolites with missing values in more than 75% of the individuals in either batch 1 or batch 2. Metabolites with missing values in 25–75% of the individuals in both batches were dichotomized as “observed” and “not observed” – referred to as “dichotomized metabolites” henceforth). Metabolites that had different missingness pattern between the batches (e.g., < 25% missing values in one batch and > 25% missing value in the second batch) were excluded. For metabolites with missing values in up to 25% of the individuals in both batches, we assumed that missing values were due to concentrations below the minimum detection limits, thus imputed the missing values for each metabolite with the lowest non-missing value of that metabolite across the sample within the batch. We then rank normalized these metabolite measures in each batch separately. In the sex-stratified analysis, we used the same rank-normalized metabolites (and did not rank-normalize within sex groups).

### Sleep disordered breathing phenotypes

We selected seven correlated phenotypes capturing potentially different aspects of SDB: Respiratory Event Index 0 (REI0), the sum of all respiratory events (apneas and hypopneas with at least 50% airflow flow reduction for a minimum duration of 10 seconds), regardless of oxygen desaturation, divided by estimated sleep time; the sum of all respiratory events associated with > = 3% oxygen desaturation divided by estimated sleep time (REI3); respiratory event duration (the average length of each respiratory event); sleep-apnea associated hypoxic burden^21^; the minimum and the average oxyhemoglobin saturation during the sleep period; and the percentage of estimated sleep period with oxyhemoglobin saturation below 90%. We then conducted sampling-weighted Principal Component Analysis (PCA), accounting for the HCHS/SOL study design, over the complete HCHS/SOL study population with non-missing SDB measures. We rank-normalized the 7 SDB measures prior to PCA analysis due the highly non-normal distribution of some of the measures and so that measures with wide range do not dominate the PCA results. We used the PCs that explain at least 10% of the variance in the SDB measures in subsequent analyses. To interpret SDB phenotypes captured by the PCs, we characterized the study populations defined by the low and high 10% values of each of the PCs selected for further analysis. Characteristics include demographic (age, sex), cardiometabolic (BMI, hypertension, diabetes), and sleep measures (SDB and self-reported insomnia, sleep duration, sleepiness) variables.

### Model covariates

All analyses used up to three conceptual models. Model 1 (i.e., base model) adjusted for demographic variables and body weight, including age, sex, field center, Hispanic/Latino background (Mexican, Puerto Rican, Cuban, Central American, Dominican, and South American and other/multi) and body mass index (BMI). Hispanic/Latino background was included because cultural differences between groups are potentially associated with differences in diet, which is highly associated with levels of many metabolites. Model 2 further adjusted for lifestyle variables – alcohol use, cigarette use, physical activity (MET-min/day), and diet (Alternative Healthy Eating Index 2010) in addition to demographic variables. Model 3 is a lifestyle and comorbidity model that is adjusted for Model 2 variables in addition to indicators for diabetes mellitus and hypertension, and continuous measures of fasting insulin, fasting glucose, HOMA-IR, HDL, LDL, total cholesterol, triglycerides, systolic blood pressure and diastolic blood pressure.

### Single metabolite associations (SMA) between individual metabolites and SDB PCs

Using survey-weighted generalized linear regressions, each metabolite’s concentration levels were regressed separately against SDB PC outcomes, with a recognition that cross-sectional data cannot establish causal direction. We used the Benjamini-Hochberg method^37^ to control false discovery rate (FDR) for multiple testing among metabolites in all models for each SDB PC in batch 1. Metabolites were flagged for further validation if the FDR corrected *p* < 0.05 in Model 1, for either SDB PC1 or PC2. In the validation analysis, we tested the associations of these flagged metabolites with SDB PCs in linear regression in batch 2 in Models 1–3. We computed one-sided p-values guided by the estimated directions of the associations in batch 1^38^, and determined replication if the one-sided p-value was < 0.05.

In a follow-up analysis, we visualized the concentrations of raw and rank-normalized metabolites from sex hormone-related pathways that are associated with SDB by gender and age strata.

### LASSO regression for constructing the SDB metabolomic risk scores (SDB-MRS)

For each SDB PC, we applied LASSO linear regression over all 582 continuously modeled metabolites, adjusted for the covariates from Model 1 (unpenalized) in HCHS/SOL batch 1. We selected the LASSO tuning parameter by minimizing the prediction error for SDB PCs in a 10-fold cross-validation. SDB-MRSs were calculated as a weighted sum of the normalized metabolite serum concentrations, with weights being the metabolite coefficients from the LASSO regression from batch 1. In association analyses using the MRSs, we standardized (z-scored) them to have mean 0 and variance 1 using the sample mean and variance **(Supplemental Table S7).**

To validate the associations between the SDB-MRS with SDB PCs, we constructed the SDB PC1-MRS and SDB PC2-MRS in batch 2 using the weights from the LASSO regression conducted in batch 1, then assessed their associations with the corresponding SDB PCs in Models 1–3. In secondary analyses we assessed potential sex differences via: (1) sex-stratified SDB-MRSs constructed based on sex-stratified LASSO; (s) sex-stratified association analyses for sex-specific and sex-combined SDB-MRSs. We also assessed the associations between SDB-MRS quartiles and the corresponding SDB PCs.

In a secondary analysis, we also constructed SDB-MRSs based on the SMA results, similar to our prior work^30^. The SMA based SDB-MRSs were weighted sums of metabolite levels where the metabolites were those with FDR < 0.05 among the metabolites modeled as continuous in the SMA batch 1 discovery analysis. The weights were the metabolite coefficients from a survey weighted unpenalized multivariate linear regression using these metabolites jointly.

### Incident outcomes

We also studied the associations of the SDB PCs and their MRSs with incident hypertension and diabetes, assessed at visit 2, among individuals free of hypertension and free of diabetes mellitus, respectively, at the baseline exam. Diabetes was derived based on American Diabetes Association (ADA) definition or self-report of diabetes mellitus. ADA criteria are based on laboratory tests - fasting glucose > = 126 mg/dL, or post-OGTT glucose > = 200 mg/dL or A1C > = 6.5%^39^. In a secondary analysis, incident diabetes was assessed separately among individuals with impaired glucose tolerance (fasting glucose within 100–125 mg/dL, or post-OGTT glucose within 140–199 mg/dL, or A1C within 5.7% − 6.5%) and among normal glycemic individuals. Hypertension was determined following the NHANES guidelines: systolic or diastolic blood pressure is greater than or equal to 140/90 or participant self-reported as currently taking antihypertensive medications^40^.

### Association analyses between SDB phenotypes and incident cardiometabolic outcomes

Finally, survey-weighted Poisson regressions were implemented to assess the associations between incident hypertension and diabetes mellitus among batch 1 and 2 combined study samples with various SDB phenotypes including benchmark singular sleep measures (i.e., REI 3%, hypoxic burden) and our newly developed composite measures (i.e., SDB PCs, and SDB-MRSs), as well as our recently developed OSA-MRS, adjusting for Model 1 and 2 covariates, respectively. The OSA-MRS was trained using LASSO on moderate to severe OSA (defined as REI3 > = 15) in the HCHS/SOL cohort and previously validated in the MESA cohort^30^. We combined the two batches in this analysis to increase statistical power by having a larger sample size. To combine metabolomics data of batch 1 and batch 2 we aggregated the metabolites from non-overlapping batch 1 and batch 2 individuals, after imputation and rank-normalization of each metabolite separately in each batch.

All analyses were done in R 3.6.3. svyglm was used for survey-weighted generalized linear regression models, and svyprcomp was used for sampling-weighted principal component analysis, both of which were from the survey package^41^. The glmnet R package (version 3.0)^42^ was used for the LASSO linear regression.

## Results

### Metabolomics sample characteristics

The main, batch 1, discovery dataset (used for SDB-SMA analysis and LASSO regression) included 1,874 female participants (mean age = 42.8), and 1,425 male participants (mean age = 41.6), and the validation dataset included 960 female participants (mean age = 51.9) and 562 male participants (mean age = 51.2) from batch 2 (Table 1). Consistent with their older age, the prevalence of moderate to severe SDB was higher in batch 2 compared to batch 1 participants (REI3≥15, 13.8% compared to 11.5% in batch 1 participants); similarly, comorbidities were higher in batch 2 participants (30.1% prevalent diabetes mellitus and 45.7% prevalent hypertension, compared to 20.4% and 32.2%, respectively, in batch 1).

### SDB PC1 and SDB PC2 characterize study population on different dimensions

**Figure 2:**
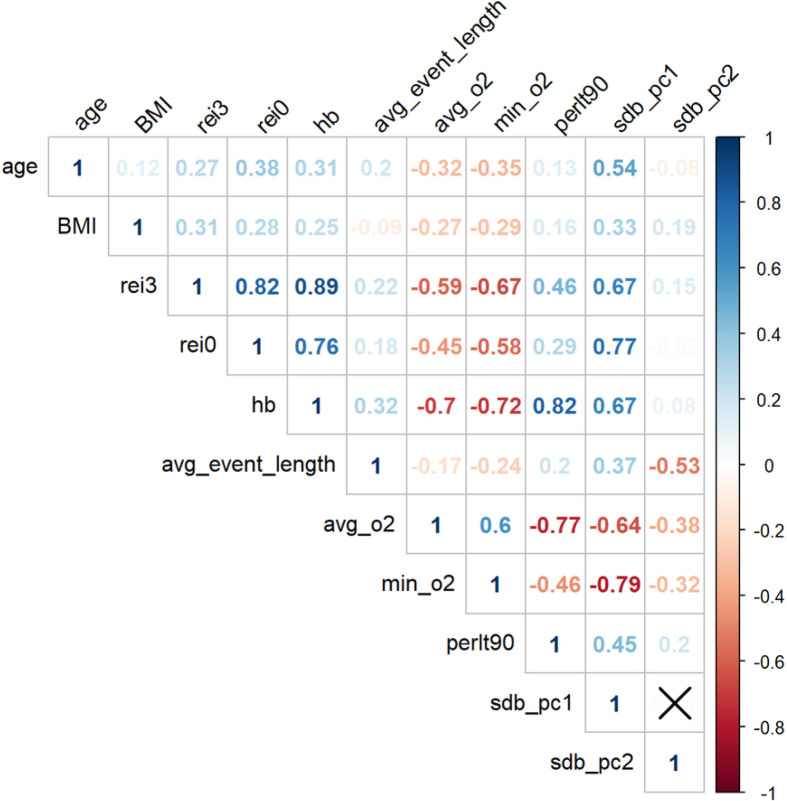
Estimated correlations between age, BMI, SDB phenotypes and SDB PCs rei3: Respiratory Event Index (REI) computed over all respiratory events, defined as apneas or hypopneas with at least 50% cannula flow reduction for a minimum duration of 10 seconds with >=3% oxygen desaturation; rei0: REI computed over all respiratory events regardless of oxygen desaturation; hb: hypoxic burden; avg_event_length: the average length of apnea and hypopnea events (combined); min_o2: minimum oxyhemoglobin saturation during sleep; avg_o2: average oxyhemoglobin saturation during sleep; perlt90: percentage of sleep time with oxyhemoglobin saturation below 90%; sdb_pc1: the first principal component of the seven sleep disordered breathing traits included in the principal component analysis; sdb_pc2: the second principal component of the seven sleep disordered breathing traits included in the principal component analysis.

In total, 11,653 HCHS/SOL study participants with complete SDB measures were included in the principal component analysis of SDB phenotypes. **Supplemental Table S1** shows the sample characteristics stratified by gender while accounting for sampling weights, so that means and proportions are representative of the HCHS/SOL target population. The first two principal components of the SDB measures accounted for 79.8% of the total variance (**Supplementary Figure S2**). For both PCs, higher values indicate more severe hypoxemia. However, PC1 is also characterized by more frequent respiratory events while PC2 is characterized by shorter respiratory events. Specifically, high SDB PC1 is correlated with increased REI3 (Spearman correlation coefficient *ρ*=0.67) and REI0 (*ρ*=0.77), increased hypoxic burden (*ρ*=0.67), high percentage of sleep time with SpO2<90% (*ρ*=0.45), decreased average oxygen saturation (*ρ*=−0.64) and lower minimum oxygen saturation (*ρ*=−0.79). High SDB PC2 is mostly correlated with reduced average event length (*ρ*=−0.53), lower average (*ρ*=−0.38) and minimum oxygen saturation (*ρ*=−0.32), and increased percentage of sleep time with SpO2<90% (*ρ*=0.2) (Fig. 2).

To better understand the phenotypic characteristics that SDB PC1 and PC2 represent, we also compared the populations defined by the top and bottom 10% of SDB PC1 and PC2 (Table 2). The top 10% compared with the bottom 10% SDB PC1 was comprised of individuals who were on average older and have a higher BMI; more likely to be male and have prevalent and incident hypertension and diabetes mellitus; and more likely to have history of smoking. The top 10% SDB PC2 compared to the bottom PC2 included participants who were slightly younger, less likely to be males, and more likely to be current smokers but did not differ in rates of baseline and incident hypertension and diabetes (Table 2). As for sleep disturbance traits, the top and bottom 10% SDB PC1 participants self-reported similar insomnia symptoms according to the Women’s Health Initiative Insomnia Rating Scale and similar sleep quality (typical night’s sleep in the past 4 weeks being restless or very restless), but reported more severe excessive sleepiness, more frequent snoring and shorter sleep duration, while the top 10% SDB PC2 participants reported worse insomnia symptoms and sleep quality, and were more likely to have long sleep ( > = 9 hours) compared to the bottom 10% SDB PC2.

### Single metabolite associations (SMA) with SDB PCs

**Figure 3:**
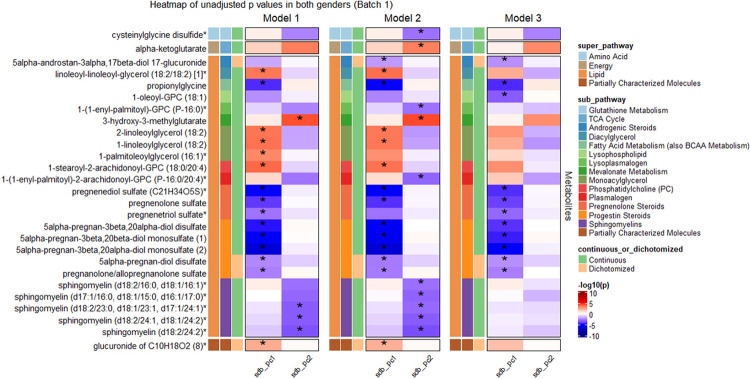
Single metabolite association analysis in both gender in batch 1 −log10(p) is based on unadjusted p derived by accounting for the complex sampling design-based degrees of freedom, using adjusted standard errors to compute the t-statistic in single metabolite association analysis with SDB PCs as dependent variables. * indicates FDR adjusted p, derived using the Benjamini-Hochberg method to control false discovery rate (FDR) for multiple testing among metabolites in all models for each SDB PC in the discovery dataset (batch 1) is below .05. sdb_pc1: the first principal component of the seven sleep disordered breathing traits included in the principal component analysis; sdb_pc2: the second principal component of the seven sleep disordered breathing traits included in the principal component analysis. Model 1 adjusted for demographic variables, including age, sex, field center, Hispanic/Latino background (Mexicans, Puerto Ricans, Cubans, Central Americans, Dominicans, and South Americans and other/multi) and body mass index (BMI). Model 2 adjusted for all model 1 covariates and lifestyle variables – alcohol use, cigarette use, physical activity (MET-min/day), and diet (Alternative Healthy Eating Index 2010) in addition to demographic variables. Model 3 adjusted for all model 1 and 2 covariates and comorbidities - indicators for diabetes and hypertension, and continuous measures of fasting insulin, fasting glucose, HOMA-IR, HDL, LDL, total cholesterol, triglycerides, systolic blood pressure and diastolic blood pressure. Metabolite with * indicates they were identified based on accurate mass data, retention time and mass spectrometry but not reference standards. Therefore, the verification is not as robust as metabolites without *.

**Figure 4:**
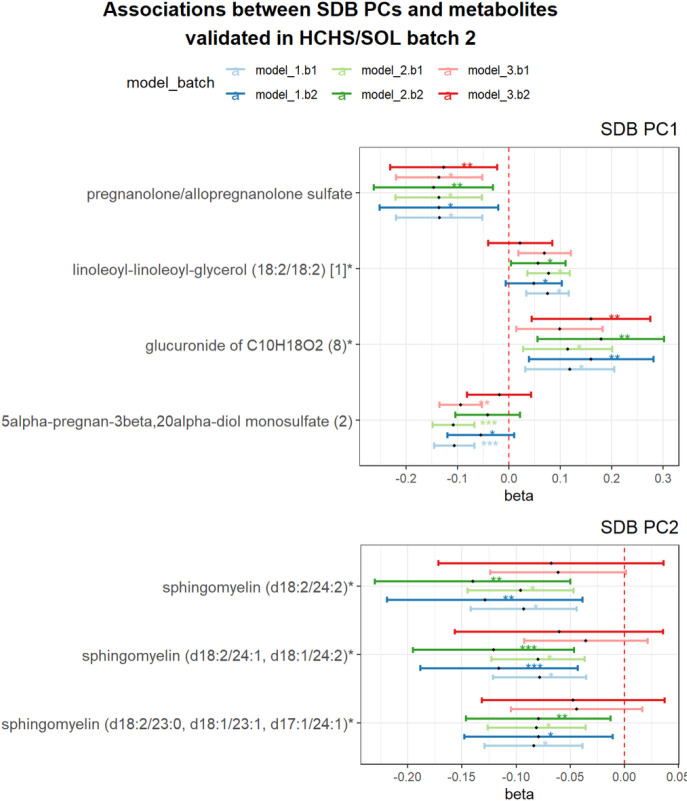
Associations between SDB PCs and metabolites replicated in batch 2 SDB PC1: the first principal component of the seven sleep disordered breathing traits included in the principal component analysis; SDB PC2: the second principal component of the seven sleep disordered breathing traits included in the principal component analysis. Model 1 adjusted for demographic variables, including age, sex, field center, Hispanic/Latino background (Mexicans, Puerto Ricans, Cubans, Central Americans, Dominicans, and South Americans and other/multi) and body mass index (BMI). Model 2 adjusted for all model 1 covariates and lifestyle variables – alcohol use, cigarette use, physical activity (MET-min/day), and diet (Alternative Healthy Eating Index 2010) in addition to demographic variables. Model 3 adjusted for all model 1 and 2 covariates and comorbidities - indicators for diabetes and hypertension, and continuous measures of fasting insulin, fasting glucose, HOMA-IR, HDL, LDL, total cholesterol, triglycerides, systolic blood pressure and diastolic blood pressure. * indicates FDR *p*<0.05 in batch 1 and one-sided *p*<0.05 in batch2. ** indicates FDR *p*<0.01 in batch 1 and one-sided *p*<0.01 in batch 2. Metabolite with * indicates they were identified based on accurate mass data, retention time and mass spectrometry but not reference standards. Therefore, the verification is not as robust as metabolites without *. b1: batch 1; b2: batch 2.

Figure 3 shows 15 metabolites associated with SDB PC1 and 4 metabolites associated with SDB PC2 (FDR P < 0.05) in HCHS/SOL batch 1 in Model 1 (the corresponding effect estimates are provided in **Supplemental Table S2**). Among the 15 SDB PC1 metabolites, four metabolites – pregnanolone/allopregnanolone sulfate, linoleoyl-linoleoyl-glycerol (18:2/18:2) [1], glucuronide of C10H18O2 (8) and 5alpha-pregnan-3beta,20alpha-diol monosulfate (2) replicated (one-sided *p*-value < 0.05) in batch 2 in Model 1 analysis (Fig. 4). Pregnanolone/allopregnanolone sulfate and glucuronide of C10H18O2 (8) remained associated with PC1 when adjusted for additional lifestyle and comorbidity covariates in batch 2. Three of the four metabolite associations with SDB PC2 in batch replicated in batch 2 (one-sided *p*-value < 0.05) in Model 1 and 2, all of which were sphingomyelin lipids - sphingomyelin(d18:2/24:2), sphingomyelin(d18:2/24:1,d18:1/24:2), and sphingomyelin(d18:2/23:0,d18:1/23:1, d17:1/24:1). Full results from the SMA sex-combined analysis are provided in **Supplemental Table S3.**

In the sex-specific SMA, tauro-beta-muricholate, a lipid from the bile acid metabolism pathway, was associated with SDB PC1 (FDR p < 0.05) among males, while no metabolite was identified for SDB PC2 in male-only analyses after FDR correction. The association of tauro-beta-muricholate with SDB-PC1 in males did not replicate in batch 2. In female-specific discovery analysis, ten metabolites were associated with SDB-PC1, of which eight were discovered in the sex-combined SMA analysis, and two, 3-hydroxyoctanoylcarnitine (1) and 3-hydroxyoctanoylcarnitine (2), were unique to the sex-stratified analysis. A single metabolite, allantoin, was associated with SDB-PC2 among females (**Supplemental Table S3**). Among the twelve metabolites identified in either the male- and female-specific SMA analysis, only the associations of pregnanolone/allopregnanolone sulfate and glucuronide of C10H18O2 (8) with PC1 were replicated in batch 2 among females (**Supplemental Table S4**). When testing for evidence of interaction with sex, only tauro-beta-muricholate had significant interaction effect (FDR *p* = 0.014) (**Supplemental Table S5**).

Given that half of the discovered and replicated SDB PC1 metabolites were from the progesterone steroids biosynthesis pathway, we compared and visualized the concentration levels of the eight progesterone steroids sulfate metabolites with statistically significant associations with SDB PC1 after FDR correction in batch 1 by age groups in each sex strata. As age increases, we observed a decreasing trend in the levels of circulating progesterone steroids sulfate metabolites in both men and women. The patterns become more visible in the rank-normalized metabolites (**Supplemental Figure S5**). Sulfated metabolites of progesterone − 5alpha-pregnan-3beta,20alpha-diol disulfate, 5alpha-pregnan-3beta,20alpha-diol monosulfate (2), and 5alpha-pregnan-3beta,20beta-diol monosulfate (1), 5alpha-pregnan-diol disulfate and Pregnanolone/allopregnanolone sulfate, were higher among younger women compared to younger men (in age groups < 40 and 40–45), while the differences diminished in older age groups (50–55, 55–60, and > 60) that would typically include post-menopausal women. The circulating pregnenolone steroids sulfate metabolites XXXpregnanediol sulfate (C21H34O5S)*, pregnenetriol sulfate*, and pregnenolone sulfate, were higher in men compared to women across all age groups. The patterns were similar in the two batches.

### LASSO regression for joint selection and estimation of metabolite associations with SDB PCs in HCHS/SOL batch 1

To identify a set of metabolites that were jointly associated with SDB PCs, we also implemented a LASSO regression in HCHS/SOL batch 1 (discovery dataset), both in sex-combined and stratified study samples. 125 metabolites were identified for SDB PC1, and 80 metabolites for SDB PC2, with 27 metabolites overlapping between the two groups. The breakdown of super pathways of the metabolites are shown in Supplemental **Figure S3** and coefficients for all metabolites from LASSO trained in sex-combined and sex-stratified samples are provided in **Supplementary Table S6**.

We constructed SDB PC1-MRS and SDB PC2-MRS for batch 1 and batch 2 HCHS/SOL participants based on results from the LASSO penalized regression. Study sample means and SD used in standardizing the MRSs are provided in **Supplemental Table S7.** In a secondary analysis, we constructed MRSs based on SMA results. **Supplemental Table S8** provides weights of these secondary SDB-MRSs. As expected by construction, all SDB-MRSs were significantly associated with their corresponding SDB PCs in batch 1 in all models. The associations replicated for both LASSO based SDB-MRSs but not for SMA-based SDB PC1-MRS in batch 2 (Table 3). Therefore, we move forward with the SDB-MRSs based on LASSO. The sex-specific SDB PC-MRSs, although also replicated in batch 2, did not show stronger associations with their corresponding SDB-PCs.

### Associations with incident cardiometabolic outcomes

In the HCHS/SOL sleep study target population, SDB PC1 showed positive associations with incident diabetes mellitus and hypertension over an average of 6.1 years (4.3–9.4 years) in both Model 1 and 2 among the samples without diabetes or hypertension at baseline, respectively. These composite phenotypes showed stronger associations than the individual SDB measures REI3 and hypoxic burden. SDB PC2 was not significantly associated with either incident outcome (**Supplemental Table S9**).

**Figure 5:**
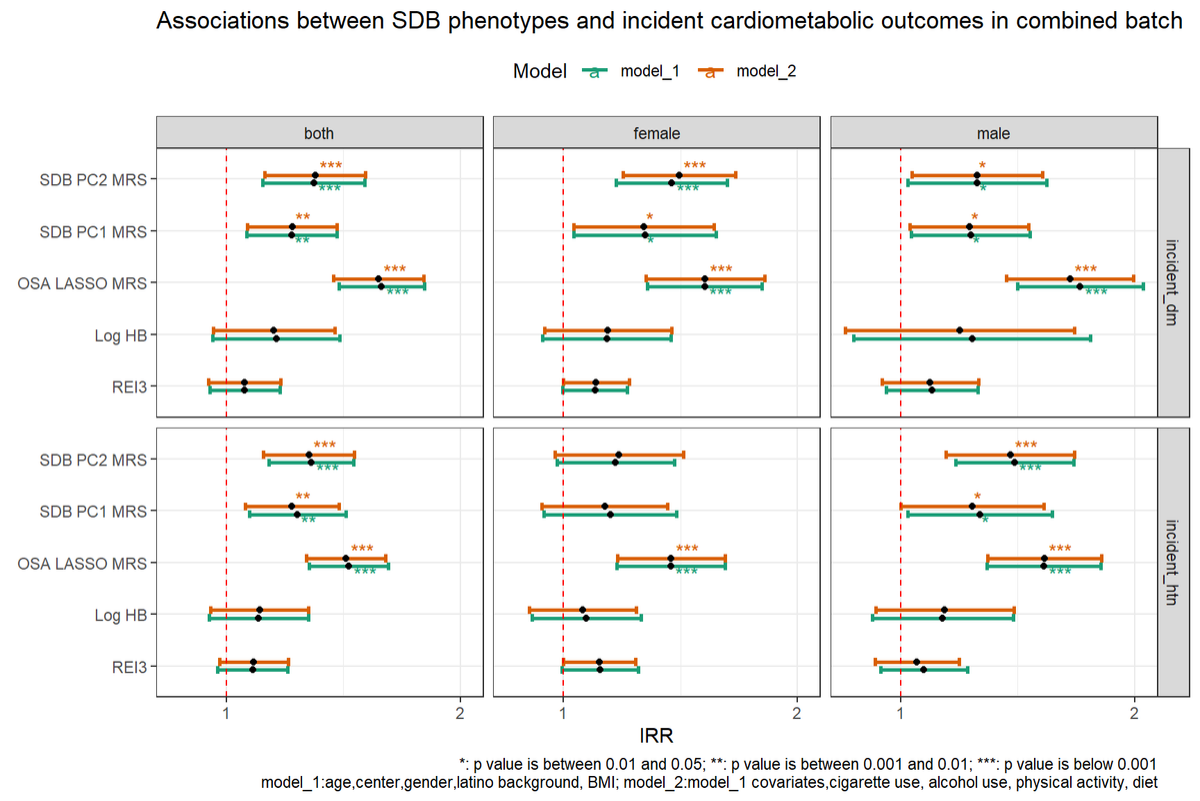
Association between SDB phenotypes and incident cardiometabolic outcomes in the combined batch * indicates p<0.05. ** indicates p<0.01. *** indicates p<0.001. Model 1 adjusted for demographic variables, including age, sex, field center, Hispanic/Latino background (Mexicans, Puerto Ricans, Cubans, Central Americans, Dominicans, and South Americans and other/multi) and body mass index (BMI). Model 2 adjusted for all model 1 covariates and lifestyle variables – alcohol use, cigarette use, physical activity (MET-min/day), and diet (Alternative Healthy Eating Index 2010) in addition to demographic variables. REI3: Respiratory Event Index (REI) computed over all respiratory events, defined as apneas or hypopneas with at least 50% cannula flow reduction for a minimum duration of 10 seconds with >=3% oxygen desaturation; HB: hypoxic burden; SDB PC1 MRS: metabolite risk score calculated based on the coefficients from LASSO regression trained in both sexes combined to predict the first principal component of the seven sleep disordered breathing traits in discovery dataset (batch 1); SDB PC2 MRS: metabolite risk score calculated based on the coefficients from LASSO regression trained in both sexes combined to predict the second principal component of the seven sleep disordered breathing traits in discovery dataset (batch 1); OSA LASSO MRS: metabolite risk score calculated based on coefficients from LASSO regression trained to predict OSA in previous publication (Zhang et al. 2022)

In the batch-combined analysis, both SDB-MRSs were significantly associated with increased incidence rate ratio (IRR) for incident hypertension, while only SDB PC2 MRS was significantly associated with developing incident diabetes mellitus, when adjusted for demographic and lifestyle risk factors (Fig. 5 **and Supplemental Table S10**). One SD increase of SDB PC2-MRS was associated with a 28% [IRR: 1.28 95% CI: 1.12–1.46, p = .0004] higher incidence rate of hypertension and a 30% [IRR: 1.30 95% CI: 1.12–1.51, p = .0005] higher incidence rate of diabetes mellitus, adjusted for demographic and lifestyle covariates (**Supplemental Table S10**). The effect estimates were slightly lower for SDB PC1-MRS when adjusting for the same covariates (Fig. 5**and Supplemental Table S10**). For comparison, we also computed OSA-MRS, 1 SD increase of OSA-MRS was associated with a 43% [IRR: 1.43 95% CI: 1.27–1.60, p < .0001] higher incidence rate of hypertension and a 57% [IRR: 1.57 95% CI: 1.38–1.80, p < .0001] higher incidence rate of diabetes mellitus. None of the single metric physiological phenotypes (i.e., REI3, HB, SDB PCs) were significantly associated with incident cardiometabolic outcomes in both models (Fig. 5**and Supplemental Table S10 – S11**)

Secondary analysis was carried out by stratifying the study samples for incident diabetes mellitus into two subgroups: individuals with normal glucose regulation (n = 1,376) and with impaired glucose regulation (n = 1,532) at baseline. The observed associations between SDB-PC1 MRS and incident diabetes mellitus became weaker in the two strata and lost statistical significance. The association between SDB-PC2 MRS was statistically significant in both groups, with a stronger association observed in the normal glycemic group (IRR = 1.42) compared to the impaired glucose regulation group (IRR = 1.25), both adjusted for demographic and lifestyle covariates. The association between OSA-MRS and incident diabetes also became weaker in both strata compared to the combined sample, and remained statistically significant only in the pre-diabetic group (IRR = 1.57 in the combined group, IRR = 1.37 in the pre-diabetic group, and IRR = 1.30 in the normal glycemic group) when adjusted for demographic and lifestyle covariates.

Focusing on OSA-MRS, which had the strongest association with incident outcomes of all MRSs, we also compared risk for incident outcomes by quartiles. Compared with the lowest quartile of the OSA-MRS, the top quartile showed more than a three-fold increase in incidence rate for diabetes mellitus [IRR: 3.98 95% CI: 2.75–5.75; p < .0001] in Model 1 and remained significant when adjusted for lifestyle covariates in addition [Model 2 IRR: 3.35 95% CI: 2.30–4.89, p < .0001] (**Supplemental Figure S4 and Supplemental Table S12)**.

There is no evidence supporting stronger associations with incident outcomes among SDB PCs-MRSs trained in each sex stratum separately versus the sex-combined MRSs.

## Discussion

We constructed new SDB measures based on seven correlated SDB phenotypes using PCA, weighted to represent the target population of the HCHS/SOL study. High scores for SDB PC1 appeared to characterize a SDB phenotype described by a high frequency of obstructive events and marked hypoxemia-a pattern typical of severe SDB and more often observed in men compared to women. In contrast, high SDB PC2 reflected a subphenotype that correlated mostly strongly with shorter event duration, and to a lesser degree, with hypoxia measures, while being almost uncorrelated with traditional event frequency measures (REI0 and REI3). In the HCHS/SOL, higher SDB PC2 was more common in younger women, individuals with more severe insomnia, self-reported poor sleep, frequent awakenings and longer sleep duration. SDB PC2 is highly correlated with shorter respiratory event duration, which has been reported in other cohorts to be more common in females, in younger individuals, and associated with higher arousal responses for any given change in oxygen saturation^43^. Moreover, in a discovery-replication approach within distinct subsamples from the HCHS/SOL, we identified multiple metabolites individually associated with each SDB PC, as well as metabolites that are collectively associated with SDB. We used the latter set of metabolites to construct MRSs of SDB aggregating multiple metabolites. The SDB-MRSs have stronger associations with incident cardiometabolic outcomes - diabetes mellitus and hypertension-compared to single SDB metrics, REI3 and hypoxic burden.

**Figure 6:**
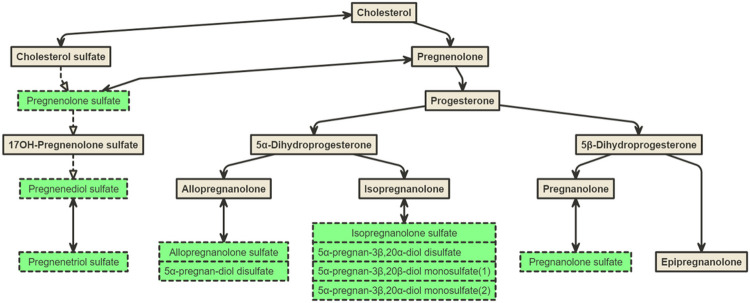
Biosynthesis of progesterone steroids Metabolites highlighted in green indicate FDR<.05 in the single metabolite association (SMA) analysis for SDB PC in the discovery dataset (batch 1) in model 1 adjusted for demographic variables, including age, sex, field center, Hispanic/Latino background (Mexicans, Puerto Ricans, Cubans, Central Americans, Dominicans, and South Americans and other/multi) and body mass index (BMI).

Higher concentrations of multiple sulfated metabolites of progesterone and its precursor pregnenolone were associated with lower (healthier) values of SDB PC1 (FDR < .05) in the discovery dataset: pregnanolone/allopregnanolone sulfate and 5alpha-pregnan-3beta,20alpha-diol monosulfate (2) (which replicated), as well as additional six progestin steroids (highlighted in green in Fig. 6). Since progesterone in circulation is quickly metabolized by the liver and has a half-life of approximately 5 minutes^44^, only the glucuronide and sulfate metabolites of progesterone steroids were measured in the Metabolon platform. Progesterone is a female reproductive hormone that is mostly synthesized in ovaries and by the placenta during pregnancy, and to a lesser degree in adrenal cortex and other tissues in both men and women, and in testes in men^45^. All progesterone steroid sulfates were present in both men and women in our dataset (**Supplemental Figure S5**). The pattern of differences in these metabolites between sexes according to age suggests that sulfated metabolites of progesterone in women are of gonadal origin, whereas the sulfated metabolites of pregnenolone are of adrenal origin. Future studies will need to verify this possibility in cohorts where the date of menopause is known. If true, these data point to the possibility that different classes of steroids of different origins may be involved in the development of SDB, and its association with incident hypertension and diabetes mellitus.

The influence of progesterone- and pregnenolone-derived steroids on SDB has been a source of interest for decades given the considerable sexual dimorphism of this trait-i.e., the prevalence, severity, and physiological subtype all vary by sex. For example, while men are 3- to 4-fold more likely to have SDB than women, this sex differences attenuates after women reach menopause^46^. Women with SDB have a less collapsible airway, more hypopneas relative to apneas, and shorter event duration than men^43^. Progesterone is a proposed mechanism for protecting women from SDB. It is an anti-oxidant^47^ that also is a respiratory stimulant that increases hypoxic and hypercapnic ventilatory response (including through effects on CO2 receptors), increases genioglossus muscle tone and decrease upper airway collapsibility^48–50^. Animal studies have shown the important roles of nuclear and membrane progesterone receptors mediating the stability of the breathing pattern and therapeutic effects in treating apnea of prematurity in both male and female mice^51^. SDB increases substantially among postmenopausal women^52–54^, which may, at least in part, relate to changes in sex hormones. Two small cross-sectional studies reported inverse between progesterone levels and OSA^53,55^. Post-menopausal women who use hormone replacement therapy that includes both estrogen and progesterone have lower respiratory event frequencies than their counterparts who do not use this therapy^56^. On the other hand, clinically induced sex hormone deficiency in young women has not associated with increased SDB^57^. The complexity of interpreting effects due to exogenous versus endogenous progesterone levels, bioavailability, receptor sensitivity, and the effects of other sex steroids has limited our understanding of role of progesterone steroids in the pathogenesis of SDB. In our study, the association with SDB PC1 suggests protective associations of progesterone steroids sulfate metabolites with SDB phenotype characterized by a high frequency of obstructive events and marked hypoxemia; while this association was observed in both women and men, this phenotype was more severe in men. Future longitudinal assessments of sex hormones and SDB may further elucidate mechanisms for SDB development across the life course.

Higher values of SDB PC2 were associated with lower concentrations of three sphingomyelins: sphingomyelin(d18:2/24:2), sphingomyelin(d18:2/24:1,d18:1/24:2), sphingomyelin(d18:2/23:0,d18:1/23:1, d17:1/24:1). Sphingomyelin has long been regarded as an inert structural component of the plasma membrane. However, recent studies have showed that it also plays an important role in the pathogenesis of cardiovascular, metabolic and neurodegenerative disease, potentially via mitochondrial dysfunction and abnormal reactive oxygen species (ROS) formation^58–61^. Dysregulation of sphingomyelin has been implicated in immune regulation, inflammation and apoptosis and acute and chronic lung pathology^62^. Several studies also reported circulating and urinary sphingolipids were altered among SDB patients and the potentials for biomarkers ^63,64^. Abnormalities in multiple lipid species have been implicated in sleep and circadian disruption^65^. Future studies are needed to understand the role of these metabolites in SDB. It should be recognized also that the SDB PCs in this study were not constructed with the goal of developing new SDB phenotypes and did not utilize comprehensive polysomnography. Future work should build on emerging literature of subtypes of SDB^19,66,67^ and characterize their metabolomics correlates and potential differences between them.

We found that metabolic scores reflecting SDB better predicted adverse cardiometabolic outcomes compared to the physiological phenotypes such as REI or those measuring hypoxia. We developed SDB-MRSs, expanding our earlier work on OSA-MRSs^30^. We now further studied the association of the MRSs with incident cardiometabolic outcomes. Previous work in HCHS/SOL demonstrated that SDB was associated with incident hypertension and diabetes and insomnia was associated with incident hypertension^20^. Other studies also have demonstrated associations between SDB with cardiometabolic and cardiovascular disorders^68,69^. Here, we saw that MRSs had stronger association with incident diabetes mellitus and hypertension compared to measured physiological traits (REIs, hypoxia-related metrics, and SDB-PCs), suggesting that the plasma-based SDB-related metabolites may be better markers of cardiometabolic risk than are physiological metrics made from a single overnight sleep study. Further, when examining only individuals with normal glycemic levels at baseline, the SDB-PC2 MRS exhibited a more robust association with incident diabetes compared to the MRS derived using a simpler OSA phenotype (a binary measure SDB) (null in the analysis). This suggests a promising role for the SDB-PC2 MRS for identifying individuals with SDB at elevated risk of developing diabetes before the onset of glucose dysregulation (i.e., early-stage diabetes). Given the null findings of many SDB intervention trials who recruited patients on the basis of physiological traits, future studies can evaluate the use of metabolic markers for identifying individuals who may benefit from SDB treatment.

Strengths of this study include the use of a large population of under-studied Hispanic/Latino adults, large panel of measured metabolites, rigorous analysis including a replication study, and assessment of association of constructed MRSs as well as of traditional SDB severity measures with incident hypertension and diabetes. The study also has a few limitations. We used PCA, a linear dimension reduction method. Despite its advantage of interpretability, it could be less flexible than other non-linear techniques, and may not be an optimal method if the underlying structure among SDB phenotypes is non-linear. Information loss may have occurred secondary to use of rank normalization of the SDB phenotypes and the metabolite levels at a preprocessing step. The discovery and the replication datasets differed by age and several health characteristics, which may have reduced the ability to replicate findings. Given the observational nature of the study, we cannot draw causal inferences. Lastly, SDB MRSs do not include all the significantly associated metabolites, including the replicated metabolite pregnanolone/allopregnanolone sulfate, because only the metabolites in continuous format can be used in the weighted sum forming the SDB MRSs.

To summarize, using a discovery-replication study design, we identified and replicated multiple metabolites associated with SDB after corrected for multiple comparisons. We constructed SDB MRSs which exhibited stronger associations with cardiometabolic sequalae of SDB, compared with physiologic SDB measures, including after accounting for demographic and lifestyle factors. These findings provide a strong basis for future evaluation of MRSs for risk stratification, and as biomarkers that guide diagnosis and treatment decisions.

## Figures and Tables

**Table 1:** Characteristics of Hispanics/Latinos represented by the metabolomic analytic sample from the HCHS/SOL study

	Batch 1			Batch 2		
Mean (SD)*	Female	Male	Overall	Female	Male	Overall
n	1874	1425	3299	960	562	1522
Age at baseline	42.82 (15.10)	41.62 (14.94)	42.22 (15.03)	51.89 (12.31)	51.18 (13.73)	51.57 (12.96)
Hispanic/Latino background (%)						
Dominican	205 (10.9)	114 (8.0)	319 (9.7)	128 (13.3)	61 (10.9)	189 (12.4)
Central American	191 (10.2)	130 (9.1)	321 (9.7)	108 (11.2)	53 (9.4)	161 (10.6)
Cuban	251 (13.4)	264 (18.5)	515 (15.6)	158 (16.5)	116 (20.6)	274 (18.0)
Mexican	753 (40.2)	531 (37.3)	1284 (38.9)	327 (34.1)	171 (30.4)	498 (32.7)
Puerto Rican	312 (16.6)	254 (17.8)	566 (17.2)	151 (15.7)	116 (20.6)	267 (17.5)
South American	113 (6.0)	86 (6.0)	199 (6.0)	74 (7.7)	40 (7.1)	114 (7.5)
Multi/other	49 (2.6)	46 (3.2)	95 (2.9)	14 (1.5)	5 (0.9)	19 (1.2)
BMI (kg/m^2^)	30.28 (6.86)	28.87 (5.37)	29.58 (6.20)	30.22 (5.96)	28.68 (5.04)	29.54 (5.62)
Alcohol drinking status (%)						
never	481 (25.7)	104 (7.3)	585 (17.7)	315 (32.9)	56 (10.0)	371 (24.4)
former	659 (35.2)	435 (30.5)	1094 (33.2)	303 (31.6)	193 (34.3)	496 (32.6)
current	733 (39.1)	886 (62.2)	1619 (49.1)	340 (35.5)	313 (55.7)	653 (43.0)
Smoking status (%)						
never	1286 (68.7)	664 (46.6)	1950 (59.1)	639 (66.6)	232 (41.4)	871 (57.3)
former	300 (16.0)	378 (26.5)	678 (20.6)	175 (18.2)	186 (33.2)	361 (23.8)
current	287 (15.3)	382 (26.8)	669 (20.3)	145 (15.1)	142 (25.4)	287 (18.9)
Physical activity (MET-min/day)	481.45 (757.57)	943.14 (1197.58)	711.22 (1027.14)	331.97 (595.88)	798.99 (1208.27)	538.38 (946.79)
The Alternate Healthy Eating Index (AHEI 2010)	46.81 (7.47)	48.87 (7.47)	47.83 (7.54)	48.31 (7.03)	50.07 (7.42)	49.09 (7.25)
OSA status = OSA, REI3 ≥ 15 (%)	141 (7.5)	240 (16.8)	381 (11.5)	94 (9.8)	116 (20.6)	210 (13.8)
REI0 (events/hr)	14.24 (16.78)	22.20 (21.53)	18.21 (19.69)	18.87 (17.42)	26.05 (21.76)	22.05 (19.78)
REI3 (events/hr)	3.82 (7.73)	8.26 (15.09)	6.03 (12.18)	5.59 (10.28)	10.03 (15.17)	7.56 (12.87)
Average length of each respiratory event (seconds)	17.82 (4.16)	19.61 (4.52)	18.71 (4.43)	18.47 (4.71)	21.06 (5.31)	19.62 (5.14)
Percentage sleep time with SpO2 < 90%	0.40 (1.50)	1.10 (4.23)	0.75 (3.18)	0.67 (2.26)	1.48 (4.13)	1.03 (3.25)
Sleep-related time in hypoxia (5% sleep < 90% saturation) (%)	42 (2.2)	82 (5.8)	124 (3.8)	32 (3.3)	36 (6.4)	68 (4.5)
Hypoxic burden (%minute/hour)	14.17 (23.38)	26.62 (44.70)	20.37 (36.17)	20.41 (30.99)	33.59 (47.14)	26.26 (39.51)
Minimum SpO2%	88.27 (5.09)	86.70 (6.18)	87.49 (5.71)	86.93 (6.11)	85.38 (6.80)	86.25 (6.47)
Average SpO2%	96.66 (0.68)	96.38 (1.09)	96.52 (0.92)	96.43 (0.82)	96.19 (1.01)	96.32 (0.91)
Baseline Diabetes status (ADA)**(%)	381 (20.3)	292 (20.5)	673 (20.4)	277 (28.9)	181 (32.2)	458 (30.1)
Baseline Hypertension status ***(%)	613 (32.7)	449 (31.5)	1062 (32.2)	439 (45.7)	256 (45.6)	695 (45.7)
Incident Diabetes (ADA) **(%)	183 (12.9)	122 (12.8)	305 (12.9)	118 (12.6)	80 (14.7)	198 (13.4)
Incident Hypertension ***(%)	172 (9.2)	127 (8.9)	299 (9.1)	137 (14.3)	85 (15.1)	222 (14.6)
Triglycerides (mg/dL)	116.38 (71.35)	144.28 (99.83)	130.28 (87.82)	135.03 (72.51)	181.31 (331.72)	155.54 (228.40)
HDL (mg/dL)	52.32 (12.79)	45.30 (12.01)	48.82 (12.89)	52.15 (12.24)	44.77 (11.20)	48.88 (12.34)
LDL (mg/dL)	119.69 (35.58)	122.57 (35.63)	121.11 (35.63)	126.35 (36.06)	122.50 (36.95)	124.67 (36.49)
Fasting glucose (mg/dL)	100.25 (32.46)	105.24 (35.22)	102.74 (33.95)	105.66 (39.89)	120.58 (56.54)	112.28 (48.54)
Fasting insulin (mU/L)	13.32 (12.18)	13.04 (19.03)	13.18 (15.96)	13.26 (9.94)	12.48 (9.19)	12.91 (9.62)
HOMA-IR	3.45 (3.80)	3.51 (5.53)	3.48 (4.74)	3.66 (3.83)	3.78 (3.44)	3.72 (3.66)
Total cholesterol (mg/dL)	195.35 (41.71)	196.42 (42.90)	195.88 (42.30)	205.41 (41.32)	200.47 (46.69)	203.22 (43.84)
Systolic blood pressure (mm Hg)	116.73 (17.69)	124.40 (15.74)	120.55 (17.18)	125.15 (20.20)	128.51 (16.48)	126.64 (18.71)
Diastolic blood pressure (mm Hg)	71.35 (10.56)	74.51 (10.99)	72.92 (10.89)	73.37 (11.22)	75.02 (10.96)	74.10 (11.13)

* Means and percentages were weighted using sampling weights to provides estimates of the HCHS/SOL target population characteristics.

** Baseline and incident diabetes are based on American Diabetes Association definition (Diabetes Care 2010;33:S62–69), defined as fasting glucose > = 126 mg/dL, or post-OGTT glucose > = 200 mg/dL or A1C > = 6.5%, or use of anti-diabetic medication.

*** Baseline and incident hypertension is defined as systolic or diastolic BP greater than or equal to 140/90 respectively, or current use of antihypertensive medications.

**Table 2:** Characteristics of study participants with low and high values of SDB PCs.

	SDB PC1		SDB PC2	
Mean (SD)*	Top10%	Bottom 10%	Top 10%	Bottom 10%
n	1166	1166	1166	1166
**Demographic variables**				
Age at baseline	54.38 (12.15)	30.80 (11.65)	39.22 (15.69)	42.23 (13.99)
Gender = Male (%)	649 (55.7)	301 (25.8)	404 (34.6)	447 (38.3)
Hispanic/Latino background (%)				
Dominican	87 (7.5)	135 (11.6)	127 (10.9)	61 (5.2)
Central American	138 (11.8)	118 (10.1)	89 (7.6)	160 (13.8)
Cuban	190 (16.3)	137 (11.8)	215 (18.4)	115 (9.9)
Mexican	439 (37.7)	479 (41.2)	390 (33.4)	542 (46.6)
Puerto Rican	203 (17.4)	171 (14.7)	250 (21.4)	146 (12.6)
South American	82 (7.0)	66 (5.7)	52 (4.5)	114 (9.8)
Multi/other	27 (2.3)	58 (5.0)	43 (3.7)	25 (2.1)
**Sleep disordered breathing**				
REI0 (events/hr)	58.19 (24.43)	2.09 (1.66)	17.25 (29.86)	17.36 (13.35)
REI3 (events/hr)	38.53 (23.53)	0.09 (0.16)	9.05 (21.60)	2.28 (2.90)
Minimum SpO2%	73.86 (7.42)	92.00 (1.54)	85.48 (7.94)	91.55 (1.83)
Average SpO2%	94.59 (1.82)	97.07 (0.33)	95.92 (1.66)	97.04 (0.29)
Percent sleep time with SpO2 < 90%	7.53 (9.40)	0.00 (0.02)	2.07 (7.07)	0.02 (0.09)
Sleep-related time in hypoxia (5% sleep < 90% saturation) (%)	466 (40.0)	0 (0.0)	118 (10.1)	0 (0.0)
Average length of each respiratory event (seconds)	23.19 (5.70)	15.72 (4.34)	14.57 (2.89)	22.98 (3.86)
Hypoxic Burden (%minute/hour)	113.89 (78.36)	1.20 (1.25)	24.92 (63.00)	14.33 (11.82)
**Lifestyle variables**				
BMI (kg/m^2^)	32.95 (5.54)	27.39 (6.71)	31.12 (7.17)	27.87 (5.22)
Alcohol drinking status (%)				
never	231 (19.8)	221 (19.0)	223 (19.2)	248 (21.3)
former	398 (34.2)	353 (30.3)	390 (33.5)	396 (34.0)
current	536 (46.0)	591 (50.7)	551 (47.3)	520 (44.7)
Smoking status (%)				
never	614 (52.7)	848 (72.8)	677 (58.2)	737 (63.4)
former	376 (32.2)	109 (9.4)	176 (15.1)	254 (21.9)
current	176 (15.1)	208 (17.9)	311 (26.7)	171 (14.7)
Physical activity (MET-min/day)	614.03 (1048.52)	696.78 (946.68)	675.35 (931.15)	727.81 (1017.02)
The Alternate Healthy Eating Index (AHEI 2010)	50.44 (7.65)	45.44 (7.44)	45.52 (6.69)	48.67 (6.48)
**Comorbidities**				
Incident Diabetes (ADA) **(%)	160 (18.6)	46 (6.3)	95 (12.0)	118 (13.1)
Baseline Diabetes status (ADA)**(%)	451 (38.7)	86 (7.4)	255 (21.9)	195 (16.7)
Incident Hypertension ***(%)	125 (10.7)	56 (4.8)	107 (9.2)	105 (9.0)
Baseline Hypertension status ***(%)	663 (56.9)	100 (8.6)	337 (28.9)	317 (27.2)
**Self-reported sleep duration and sleep disturbance**				
Sleep duration (hours)	7.81 (1.38)	8.37 (1.45)	7.91 (1.55)	7.82 (1.13)
Women’s Health Initiative Insomnia Rating Scale (WHIIRS) total score	6.43 (5.41)	6.48 (5.08)	7.58 (5.33)	5.70 (5.02)
Typical nighťs sleep in past 4 weeks (restless or very restless) (%)	214. (20.5)	208. (20.7)	256. (24.7)	187. (18.2)
Take sleeping pills (%)	83. (7.2)	52. (4.5)	115. (10.1)	76. (6.6)
Trouble getting back to sleep (3 or more times a week) (%)	215. (18.9)	193. (17.0)	252. (22.4)	214. (18.9)
Wake up earlier than you plan (3 or more times a week) (%)	269. (23.3)	235. (20.5)	259. (22.7)	256. (22.2)
Wake up several times at night (3 or more times a week) (%)	487. (42.2)	328. (28.6)	426. (37.3)	383. (33.1)
Trouble falling asleep (3 or more times a week) (%)	249. (21.6)	262. (22.9)	334. (29.3)	263. (22.8)
Epworth Sleepiness Scale (ESS) total score	7.21 (5.51)	5.67 (4.21)	5.35 (4.47)	5.37 (4.51)
Epworth Sleepiness Scale (ESS) total score > = 10 (%)	247. (21.5)	135. (11.8)	165. (14.5)	163. (14.1)
Self-reported snoring (6–7 nights a week) (%)	627. (65.7)	67. (8.5)	249. (29.2)	200. (25.2)
**Heart rate during sleep**				
Minimum resting heart rate during sleep (beats per min, BPM)	50.18 (9.64)	51.00 (7.72)	52.23 (9.05)	51.86 (7.84)
Maximum resting heart rate during sleep (BPM)	101.05 (12.74)	101.81 (13.67)	101.32 (13.39)	97.32 (10.37)
Average resting heart rate during sleep (BPM)	70.28 (9.40)	67.41 (8.95)	70.68 (9.65)	66.26 (9.70)
Standard deviation of resting heart rate during sleep	5.84 (1.99)	5.35 (1.44)	5.48 (1.70)	4.96 (1.12)

SDB: sleep disordered breathing; PC: principal component; ESS: Epworth Sleepiness Scale; OSA: moderate or severe OSA (REI3 ≥ 15)

* Means and percentages were weighted using sampling weights to provides estimates of the HCHS/SOL target population characteristics.

** Baseline and incident diabetes are based on American Diabetes Association definition (Diabetes Care 2010;33:S62–69), defined as fasting glucose > = 126 mg/dL, or post-OGTT glucose > = 200 mg/dL or A1C > = 6.5%, or use of anti-diabetic medication.

*** Baseline and incident hypertension is defined as systolic or diastolic BP greater than or equal to 140/90 respectively, or current use of antihypertensive medications.

**Table 3:** Estimated associations between SDB PC metabolite indices and their respective phenotypes, in batch 1 and 2

	Batch 1						Batch 2					
	Both		Female		Male		Both		Female		Male	
	Coefficient	p	Coefficient	p	Coefficient	p	Coefficient	p	Coefficient	p	Coefficient	p
SDB PC1 MRS												
Model 1	0.29 [.24,.34]	2.03E-33	0.30 [.25,.36]	1.55E-27	0.26 [.19,.33]	1.44E-12	0.15 [.08,.23]	**1.15E-04**	0.09 [.00,.19]	0.0611	0.20 [.10,.31]	**1.36E-04**
Model 2	0.29 [.24,.34]	4.75E-34	0.31 [.25,.36]	2.72E-28	0.26 [.19,.33]	9.72E-13	0.15 [.07,.23]	**2.23E-04**	0.08 [−.01,.17]	0.0914	0.20 [.10,.30]	**< 0.0001**
SDB PC2 MRS												
Model 1	0.23 [.19,.28]	6.04E-22	0.24 [.16,.32]	7.71E-10	0.23 [.17,.28]	1.12E-14	0.14 [.05,.22]	**1.40E-03**	0.14 [.01,.26]	**0.0353**	0.14 [.05,.23]	**2.15E-03**
Model 2	0.23 [.18,.28]	5.5E-22	0.23 [.15,.30]	1.2E-09	0.22 [.17,.28]	2.35E-15	0.14 [.06,.22]	**3.97E-04**	0.13 [.02,.24]	**0.0228**	0.15 [.06,.25]	**2.26E-03**
Sex Specific SDB PC1 MRS												
Model 1			0.32 [.26,.37]	1.94E-32	0.32 [.26,.38]	2.82E-26			0.08 [.00,.15]	**0.0457**	0.13 [.04,.21]	**2.09E-03**
Model 2			0.32 [.27,.37]	5.74E-35	0.32 [.26,.37]	8.5E-28			0.07 [−.01,.14]	0.0758	0.13 [.04,.21]	**2.38E-03**
Sex Specific SDB PC2 MRS												
Model 1			0.13 [.06,.20]	0.000112	0.23 [.17,.30]	2.77E-12			0.03 [−.09,.14]	0.6426	0.12 [.02,.22]	**1.89E-02**
Model 2			0.10 [.04,.17]	0.001702	0.22 [.16,.29]	4.38E-12			0.04 [−.06,.13]	0.4718	0.13 [.04,.22]	**5.27E-03**
SMA SDB PC1 MRS												
Model 1	0.17 [.12,.21]	2.72E-13	0.17 [.12,.23]	3.62E-10	0.15 [.08,.21]	5.91E-06	0.02 [−.04,.09]	5.15E-01	0.05 [−.04,.14]	0.2409	-0.03 [−.14,.07]	5.40E-01
Model 2	0.17 [.12,.21]	6.99E-14	0.17 [.12,.22]	1.55E-11	0.15 [.09,.21]	2.02E-06	0.02 [−.05,.08]	5.89E-01	0.04 [−.04,.13]	0.3040	-0.03 [−.14,.07]	5.28E-01
SMA SDB PC2 MRS												
Model 1	0.13 [.09,.18]	1.08E-08	0.13 [.07,.20]	5.37E-05	0.13 [.07,.20]	6.25E-05	0.09 [.03,.16]	**4.78E-03**	0.10 [.01,.19]	**0.0225**	0.08 [−.01,.18]	8.69E-02
Model 2	0.13 [.09,.18]	1.03E-08	0.13 [.07,.20]	7.95E-05	0.13 [.07,.20]	5.65E-05	0.10 [.04,.17]	**1.70E-03**	0.11 [.03,.20]	**0.0107**	0.10 [.01,.19]	**3.90E-02**

* per 1 STD increase in the metabolite index (MRS).

Model 1 adjusted for demographic variables, including age, sex, field center, Hispanic/Latino background (Mexicans, Puerto Ricans, Cubans, Central Americans, Dominicans, and South Americans and other/multi) and body mass index (BMI); Model 2 adjusted for all model 1 covariates and lifestyle variables – alcohol use, cigarette use, physical activity (MET-min/day), and diet (Alternative Healthy Eating Index 2010) in addition to demographic variables.

SDB PC1 MRS: metabolite risk score calculated based on the coefficients from LASSO regression trained in both sexes combined to predict SDB PC1 in discovery dataset (batch 1); SDB PC2 MRS: metabolite risk score calculated based on the coefficients from LASSO regression trained in both sexes combined to predict SDB PC2 in discovery dataset (batch 1);Sex Specific SDB PC1 MRS: metabolite risk score calculated based on the coefficients from LASSO regression trained in each sex strata to predict SDB PC1 in discovery dataset (batch 1); Sex Specific SDB PC2 MRS: metabolite risk score calculated based on the coefficients from LASSO regression trained in each sex strata to predict SDB PC2 in discovery dataset (batch 1);SMA SDB PC1 MRS: metabolite risk score calculated based on coefficients from unpenalized regression of metabolites identified in single metabolite association analysis with SDB PC1 in discovery dataset (batch 1);SMA SDB PC2 MRS: metabolite risk score calculated based on coefficients from unpenalized regression of metabolites identified in single metabolite association analysis with SDB PC2 in discovery dataset (batch 1)

## Data Availability

HCHS/SOL data are available through application to dbGaP according to the study specific accessions. HCHS/SOL phenotypes are available in: phs000810. HCHS/SOL genotyping data: phs000880. HCHS/SOL metabolomics data are available via data use agreement with the HCHS/SOL Data Coordinating Center at the University of North Carolina at Chapel Hill, see collaborators website: https://sites.cscc.unc.edu/hchs/.
